# Comprehensive Analysis of Soybean Mosaic Virus P3 Protein Interactors and Hypersensitive Response-Like Lesion-Inducing Protein Function

**DOI:** 10.3390/ijms20143388

**Published:** 2019-07-10

**Authors:** Hexiang Luan, Wenlin Liao, Haopeng Niu, Xiaoyan Cui, Xin Chen, Haijian Zhi

**Affiliations:** 1 National Center for Soybean Improvement, Nanjing Agricultural University, Nanjing 210095, China; 2 Institute of Vegetable Crops, Jiangsu Academy of Agricultural Sciences, Nanjing 210014, China

**Keywords:** soybean, soybean mosaic virus, P3, cDNA library screening, hypersensitive response like lesion inducing

## Abstract

Soybean mosaic virus (SMV) is one of the most prevalent and important pathogens of soybean, which produces 11 proteins, and the third protein, P3, was suggested to be involved in virus movement and replication, as well as host infection. During the virus infection, host proteins are essential in the virus cycle. However, there is no comprehensive report on the network of host proteins that interact with P3. Fifty-one interactors were identified by using the P3 protein as the bait against the SMV SC15 strain-challenged soybean cDNA library. These proteins were classified into five groups, including transport and protein transport-related proteins, defense and disease-related proteins, photosynthesis proteins, cellular metabolic proteins, and unknown proteins. Among these proteins, the protein defined as hypersensitive response-like lesion-inducing (HRLI) appeared multiple times and showed strong affinity with P3, which indicated its important role in SMV infection. Thus, it was chosen for further investigation. Phylogenetic classification showed that paralog proteins GmHRLI-1 and GmHRLI-2 clustered together and shared 90% homologous identity. Bimolecular fluorescence complementation (BiFC) assay was carried out to confirm the interaction, and fluorescence was detected at the cell periplasmic as well as at the nucleus. Subcellular localization showed that GmHRLI was localized to the cell periplasmic, while the co-localization of GmHRLI and P3 signals was also observed in the nucleus, suggesting that GmHRLI could interact with P3 and promoted the translation of P3 to the nucleus. Moreover, the gene expression of GmHRLI was abundant in the roots, leaves, and flowers, and could be induced by SMV infection, suggesting its involvement in SMV infection. Our results together lay the foundation to explore the mechanisms of P3 in the HR process and the HRLI protein function in SMV response.

## 1. Introduction

Soybean is an important resource of oil all over the world. The *potyvirus soybean mosaic virus* (SMV) is one of the most widely distributed viral pathogens and an important disease that affects soybean [Glycine max (L.)] production [1]. SMV infection has been reported to cause 15–30% loss of yield [2]. The virus is transmitted primarily by the aphid vector, and there are reports of seed transmission as well [3,4]. SMV infection results in leaf wrinkling, mosaic symptoms, and necrosis in soybean, and reduces the chlorophyll content in the chloroplast. Based on the phenotypic reaction on a set of differential soybean cultivars, SMV isolates have been classified into seven strains in North America (G1–G7) [5,6], and 21 strains in China (SC1-SC21) [7,8]. Genetic mapping has identified three resistance genes (*Rsv1*, *Rsv3*, *Rsv4*) that provide resistance against North American strains and a series of genes (*Rsc3*, *Rsc4*, *Rsc8*, *Rsc14*) that provide resistance against strains from China [9,10,11,12].

The SMV genome comprises a single-stranded, positive sense [ss(+)] RNA genome ~10 kb in size, which is translated to a single polyprotein. Then, the polyprotein is cleaved by viral-encoded proteases to generate 10 mature proteins: P1, helper component-proteinase (HC-Pro), P3, 6K1, cylindrical inclusion (CI), 6K2, viral protein genome-linked (VPg), nuclear inclusion a proteinase (NIa-Pro), nuclear inclusion b (NIb, RNA-dependent RNA polymerase), and coat protein (CP) [13,14]. Additionally, ribosome slippage has been shown to generate the P3N-PIPO peptide [15,16]. Of the 11 potyvrial proteins, most of the proteins are multifunctional. Virus survival in the host recruits plant proteins, which facilitates viral infection, including cell-to-cell movement, long distance movement, and replication in plants. Accumulating evidence indicates that CP, VPg, HC-Pro, CI, and P3N–PIPO play a role in intercellular transport [17,18,19,20,21,22,23]. Based on the previous study, HC-Pro and VPg are involved in viral genome replication or RNA binding progress [24,25]. The potyvirus CI has been suggested to play a role in cell-to-cell movement [26,27]. Although the mechanism of potyviral long-distance movement is poorly understood, the CP, HC-Pro, VPg, and 6K2 have been shown to be implicated in long-distance movement [28,29,30,31]. Both the P3 and Hc-Pro proteins are virulence determinants of the *Rsv1*-genotype soybean plant [32,33]. Also, the CI protein is involved in virulence determination in the *Rsv4*-genotype soybean plant [34]. As for the potyvirus, numerous host proteins have been shown to contribute to various aspects of viral infection in the plant. Previous studies have shown that the P3 protein of SMV is associated with the endoplasmic reticulum (ER) membrane and interacts with the eukaryotic elongation factor 1A (eEF1A) protein, which is essential for SMV survival in the plant [35]. Another interactor of P3 is RbCS (RuBisCO small subunit), which is supposed to contribute to symptom development [36]. P3 was also found to interact with actin-depolymerizing factor 2, which might play a role in virus movement [37]. P3 has a connection with the potyvirus encoded protein P1 [38]. However, the precise function of P3 has been poorly elucidated until now.

Due to the devastating effort of SMV on soybean, it is of great significance to understand the network of the soybean genes involved in SMV infection. Based on the proteins that interact with P3, the protein with the strongest affinity and highest frequency was worthy for further characterization. Here, we screened for host proteins that interacted with the P3 protein of SMV using a yeast two-hybrid screen against a soybean cDNA library constructed from SMV-infected leaves. This study focuses on theHRLI encoded by the *Glyma19G163200* gene. BiFC confirmed the interaction between GmHRLI and P3, and the gene expression of GmHRLI suggested its role in SMV infection.

## 2. Results

### 2.1. Construction, Validation, and Auto-Activation of the pBT3-P3 Bait Vector

**Figure 1:**
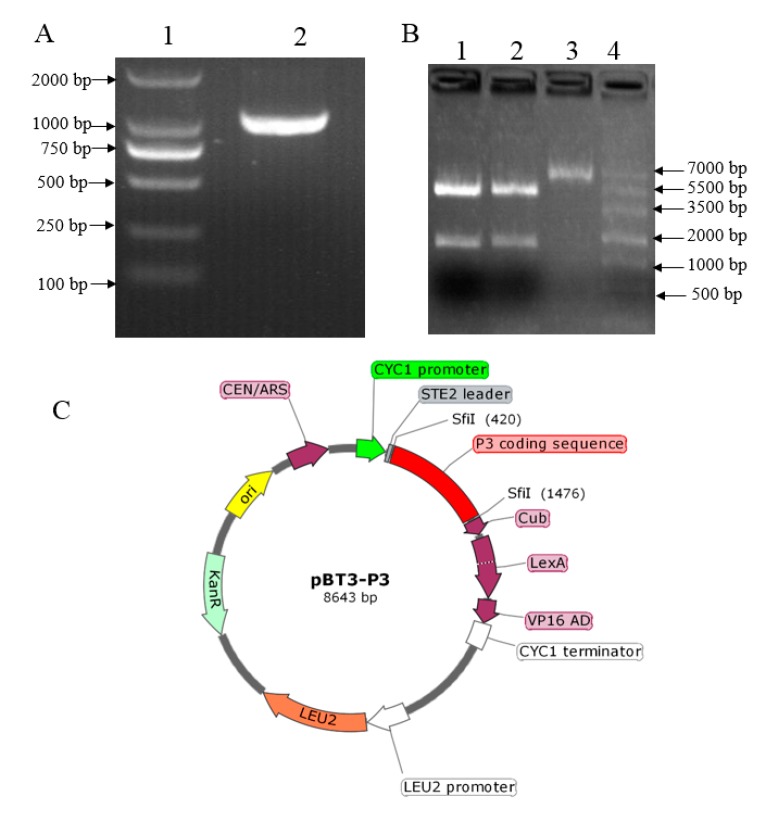
Construction and verification of the bait vector. (**A**) Gel electrophoresis image of P3 amplification from cDNA. Lane 1 is a DNA marker in which the strip size was written on the left, and lane 2 is the PCR product of P3 from cDNA. (**B**) Gel electrophoresis image of the digested recombinant plasmid. lane 1 and 2: pBT3-P3 digested by *Sfi* I; lane 3: pBT3 plasmid; lane 4: marker in which the strip size was indicated on the right. (**C**) The image of the plasmid pBT3-P3 construction.

The SMV P3 was amplified from cDNA produced from the soybean leaf tissue inoculated with the soybean SC15 strain using the primer with *Sfi* I endonuclease sequence (Figure 1A). The bait plasmid pBT3-P3 was generated by ligation of both P3 and pBT3 digested by *Sfi* I. The recombinant vector was validated by restriction enzyme digestion, which showed that the P3 fragment was released from the construct after *Sfi* I digestion (Figure 1B), indicating that the P3 was inserted into the bait vector. Additional confirmation was obtained by sequencing the recombinant clone, which showed 100% alignment with the sequence from the soybean genome, to confirm the presence of the P3 insert. The image of the plasmid pBT3-P3 construction showed the P3 was inserted between *Sfi* I (Figure 1C). Taken together, these results indicated that the P3 fragment was constructed in the bait vector successfully.

In order to check whether the bait vector displays any auto-activation of growth reporter genes adenine (Ade) and histidine (His), the pBT3-P3 was transformed together with the empty prey vector pPR3-N-R1R2. The yeast cells containing both plasmids were selected on synthetically defined medium lacking leucine (Leu), tryptophan (Trp), His, and Ade (SD^-Leu-Trp-His-Ade^) plates. No colonies were observed growing under these nutrient selection conditions, indicating that the pBT3-P3 construct did not auto-activate the selection markers and could be used for library screening.

### 2.2. Selection of SMV P3 Interactors Using Yeast Two Hybrid Analysis

In order to identify novel potential soybean interactors of SMV P3, the pBT3-P3 was co-transformed with the soybean cDNA library, which was generated from SMV-infected plants into yeast NMY51 cells. Transformants were selected on SD^-Leu-Trp^ plates to ensure that both bait and prey vectors were contained in yeast cells. The initial screening yielded 6.8 × 10^6^ transformants, and the transformation efficiency was calculated to be 0.24 × 10^6^, based on the number of colonies on SD^-Leu-Trp^ plates. Colonies containing both vectors were transferred on the SD^-Leu-Trp-His-Ade^ plates, and only the colonies that grew well were considered as potential positive interactors.

**Figure 2:**
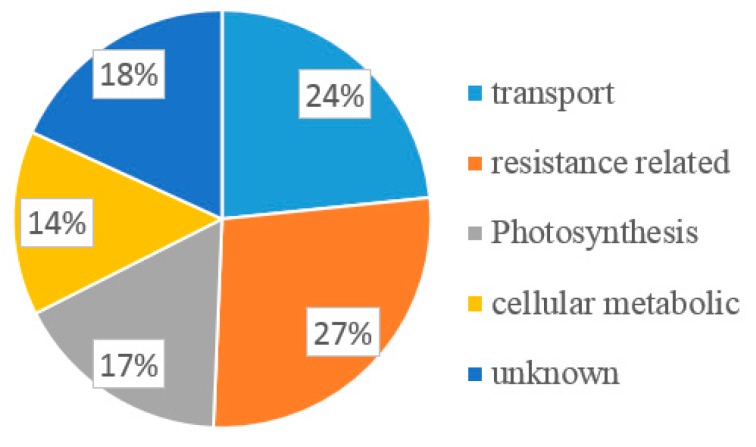
Function classification of all the potential candidate interactors of P3 by their putative function blasted in plant.

The positive colonies were randomly selected for colony PCR detection by sequencing primer pPR3-N F and R. Only colonies with a ~1 kb insert size showed on agarose gel were considered to be positive, and 120 colonies were selected for sequencing. All the sequences were analyzed using BLAST online. Finally, 77 ESTs encoding 51 potential interactors were identified (Table S1). The total of 51 interacting proteins were categorized according to their potentially functional attributes as transport, protein transport, defense, resistance related, photosynthesis, basal function, and unknown. The color-coded pie chart showed the percentage of proteins that interact with P3 from each category (Figure 2). Of the proteins identified from the 51 colonies, 24% were transport-related proteins such as zinc transporter, mitochondrial import inner membrane translocase, subunit Tim17/22 (Tim), Sec-independent protein translocase protein (TatC), and plant-specific mitochondrial import receptor subunit TOM20 (TOM20). More than one-quarter (27%) of the candidate proteins were related to resistance, such as (eEF1A [35], Pathogenesis-Related 1a (PR1a) precursor, actin-depolymerizing factor 2 (ADF2) [37], HRLI, and ubiquitin-conjugating enzyme 32 (UBC32). There were also proteins belonging to the photosystem group (17%), such as photosystem II subunit X (PSBX), cytochrome B5 isoform 1 (CYB5), tetratricopeptide repeat (TPR)-like, and copper response defect1 (CRD1). Proteins related to cellular metabolism (14%) were also identified, such as 60S ribosomal protein L18a, metallothionein 2B (MT2B), ubiquitin-conjugating E2 (UBC2), and selenoprotein-related (SELT). The remaining 18% of proteins were uncharacterized. From the 51 interactors, 18 colonies were chosen as candidates based on their potential function related to resistance or pathogens infection.

**Figure 3:**
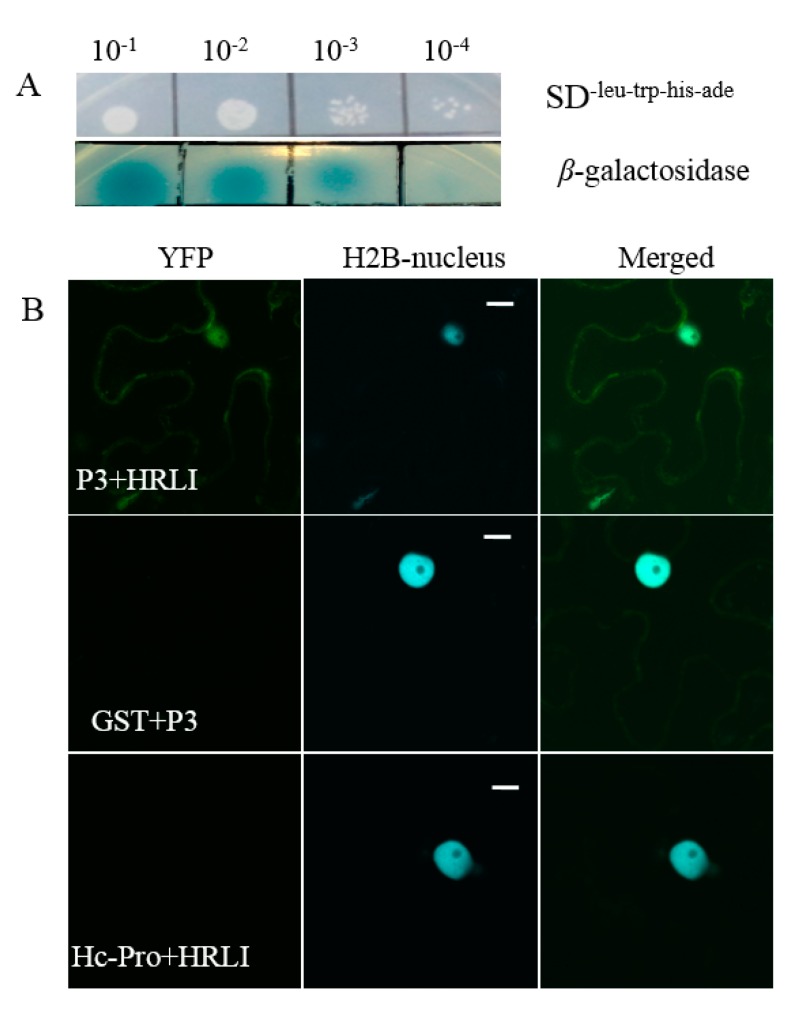
Interaction of soybean mosaic virus (SMV-P3) and the GmHRLI protein. (**A**) Confirmation of the interaction of P3 with candidate interactors in yeast NMY51 cells, and the interaction intensity shown by tipping different concentrations of interaction solution. Each result was representative of three biological repeats. (**B**) The interaction of GmHRLI and P3 shown in *Nicotiana benthamiana*. Transgenic *Nicotiana benthamiana* expressing CFP-H2B (nuclear localized histone 2B) were used for theBiFC assays. Scal bar = 10 μm. GST and Hc-Pro were used as control, respectively. Images are representative of three separate infiltrations from two independent experiments for each interaction.

To further confirm the interaction, 18 candidate interactors were selected with the putative function related to host resistance or virus infection. All 18 candidates were co-transformed with empty bait vector pBT3 into the yeast strain NMY51 by the lithium acetate method, and no colony was observed on the SD^-Leu-Trp-His^ medium, which indicated that the candidates could not automatically activate the reporter genes (Figure S1). The 18 candidate interactors were each co-transformed with P3 into NMY51 cells, and the transformants were selected on SD^-Leu-Trp-His -Ade^ plates with X-α-gal (Figure S2). Among the 18 proteins, candidate No.3-3, the HRLI protein, showed strong interaction with P3 on selection plates at 10^−1^, 10^−2^, 10^−3^, and 10^−4^ dilution (Figure 3A, upper panel), and was chosen for further investigation. The cells that contained both HRLI and P3 were diluted 10, 100, 1000, and 10,000 times in 0.9% NaCl and plated on SD^-Leu-Trp-His-Ade^ X-α-gal plates. The results showed GmHRLI and P3 had strong binding affinity at dilution ratios of 1:10, 1:100, and 1:1000, and weak affinity at 10,000 times (Figure 3A, lower panel).

### 2.3. Verification of the Interaction between GmHRLI and P3 Using BiFCAssay

Next, we confirmed the interaction of the full-length GmHRLI protein with P3 of SMV using BiFC assay. Proteins were fused with N-\C-terminal halves of the yellow fluorescent protein (YFP) and co-infiltrated in *Nicotiana benthamiana*. The protein interactions combine the two halves of YFP and show positive interaction with fluoresence under a laser scanning confocal microscope. Confocal microscope analysis detected the interaction of the HRLI protein and P3 in the cell periphery as well as the nucleus (Figure 3B, top panel). In contrast, P3 did not interact with the soybean GST (glutathione-S-transferase) (Figure 3B, middle panel). Conversely, the GmHRLI protein did not interact with the SMV HC-Pro protein (Figure 3B, bottom panel). Together, these results confirmed that the GmHRLI protein interacted with SMV P3 specifically.

### 2.4. Sequence Analysis of HRLI Genes

**Figure 4:**
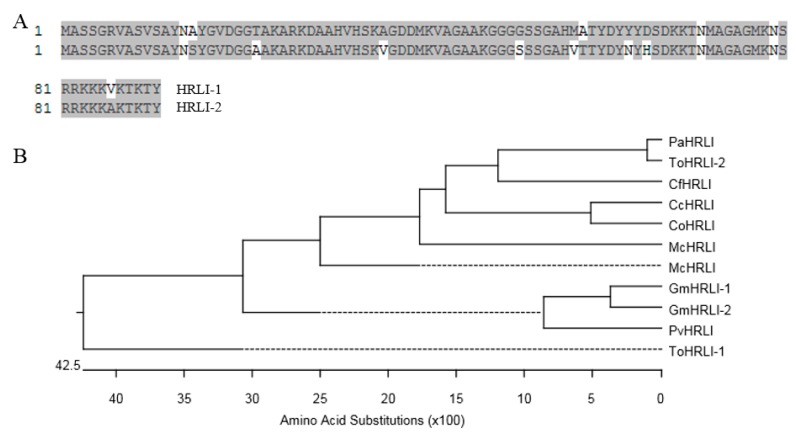
Sequence analysis of HRLI proteins. (**A**) Amino acid sequence alignment of GmHRLI paralog proteins, which were carried out using ClustalW in the Megalign program of the DNASTAR package, with identical residues shaded in gray. Numbers indicate the position of amino acid residues. (**B**) Phylogenetic relationships of HRLI from different species which were carried out using ClustalW in the Megalign program of the DNASTAR package. The abbreviation species are as following: Pv (*Phaseolus vulgaris*), Mc (*Macleaya cordata*), Cf (*Cephalotus follicularis*), To (*Trema orientale*), Pa (*Parasponia andersonii*), Cc (*Corchorus capsularis*), and Co (*Corchorus olitorius*).

There are two isoforms of *HRLI Glyma19G163200* (*GmHRLI-1*) and *Glyma03G161600* (*GmHRLI-2*) in the *Glycine max* genome, the predicted protein sequences of which shared ~90% identity with each other (Figure 4A). Multiple amino acid sequence alignment and phylogenetic analysis showed that the GmHRLI-1 and GmHRLI-2 clustered together and had a close relationship with PvHRLI (Figure 4B).

**Figure 5:**
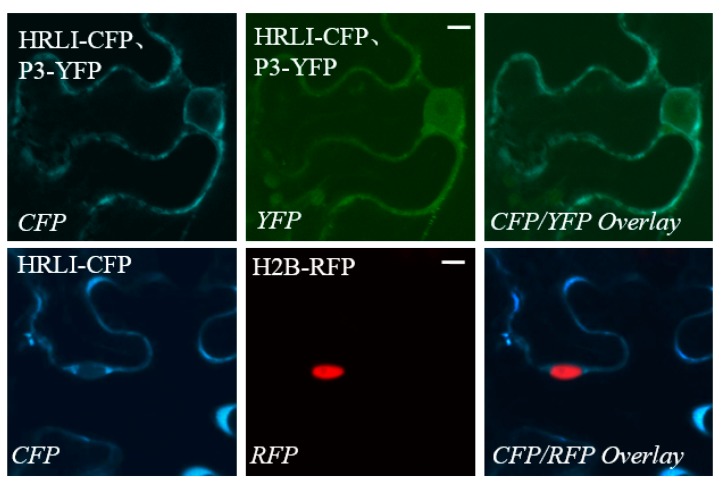
Localization of GmHRLI and co-localization with P3. C-terminal yellow flourescent protein (YFP)/CFP-tagged proteins were transiently expressed individually (GmHRLI-CFP, P3-YFP) or co-expressed (P3-YFP, GmHRLI-CFP) in *N. benthamiana* via agroinfiltration. Scale bars = 10 µm. GmHRLI-CFP was expressed in transgenic plants expressing RFP-H2B (nuclear localized). Bottom italicized letters indicate the fluorescent channel used for imaging. Images are representative of three separate infiltrations from two independent experiments for C-terminal tagged flourescence proteins.

The GmHRLI protein contained a conserved HR-like lesion-inducing domain from M1 to the M138 amino acid and a DoxX domain from F6 to the P100 amino acid by the MOTIF Search online. GmHRLI is predicted to be a chloroplast transit protein (PlantLoc probability: 1.0 http://cal.tongji.edu.cn/PlantLoc/index.jsp). Indeed, transiently expressed GmHRLI were primarily localized in the cell periphery (Figure 5, lower panel). Our previous result showed that P3 localized to the ER, but not in the nucleus [35]. However, the localization of P3 proteins was inconsistent with the BiFC result that showed interaction in the cell periphery as well as in the nucleus (Figure 3B top panel). To address this, we assayed localization in leaves co-expressing both GmHRLI and P3 proteins. Interestingly, the co-expression of GmHRLI and P3 resulted in the cell periphery localization of GmHRLI and cell periphery as well as the nucleus localization of P3 (Figure 5, upper panel). Together, these results suggested that GmHRLI promoted P3 re-localization to the nucleus through their interaction.

### 2.5. Expression Patterns of GmHRLI Gene

**Figure 6:**
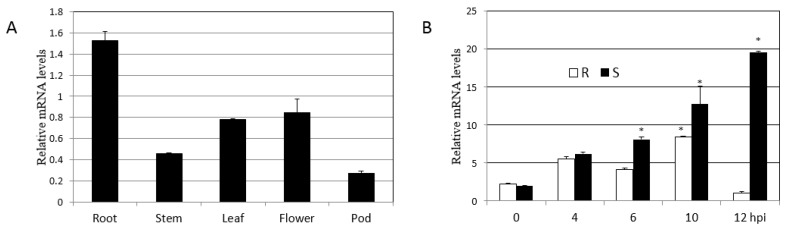
Expression profiling of *GmHRLI-1* in soybean by qPCR. (**A**) Relative mRNA level of *GmHRLI-1* in different indicated tissues of the wild-type soybean plant. (**B**) Relative mRNA levels of *GmHRLI-1* gene in R (resistance) and S (susceptible) soybean leaf tissue after SMV infection at 0, 4, 6, 10, and 12 hpi (hours post-inoculation). Error bars indicate SD (n = 3). Asterisks denote significant difference from the 0 time point, as determined by the *t*-test, *P* < 0.001. Each result is representative of three biological repeats.

We next used qPCR analysis to determine the expression pattern of the gene encoding the soybean HRLI protein. First, we analyzed different tissues of soybean to monitor *GmHRLI* transcript levels. qPCR analysis showed the maximum abundance of GmHRLI-1 in the root and also high expression in leaf and flower tissues (Figure 6A). Then, we analyzed *GmHRLI* expression in SMV-infected plants. Both resistance and susceptible soybean plants were inoculated with SMV, and the expression of the *GmHRLI* gene was assessed at various time points. Our results showed the induction of this gene in both resistance and susceptible plants. However, susceptible plants showed a higher induction of the gene at all the time points compared to resistant plants (Figure 6B). These results suggested that *GmHRLI* gene expression was induced by SMV infection, and that higher expression induction in susceptible plants indicated possible involvement in the resistance response.

## 3. Discussion

In this study, we used a yeast two-hybrid system with the SMV-P3 protein as the bait to screen the soybean cDNA library, and aimed at identifying proteins that interacted with SMV-P3. Previous studies showed that P3 is important for virus infection and movement [39,40,41], as well as the virulence determinant of the Rsv1 and Rsv4-genotype soybeans [32,42,43,44]. Based on the predicated functions of 77 ESTs, 18 ESTs were classified together and involved in transport progress, 21 ESTs were resistance related, 13 ESTs participated in the photosynthesis pathway, 11 ESTs were related to cellular metabolism, and 14 ESTs were of unknown function. However, some of the candidate proteins showed high homology, and a low ratio of cloned sequence/full length indicated the false positive in yeast. The BLAST results showed that most of the candidate proteins are related to movement and resistance, which is correlated to the function of P3. Some of the candidates have been reported before, including the ADF2 protein, which is an essential host factor for SMV-P3 in the process of SMV infection [35]. The P3 targets host elongation factors and results in the unfolded protein response (UPR), which in turn facilitates SMV replication and places eEF1A upstream of the binding protein (BiP) in the ER stress response during pathogen infection [35]. The further characterization of other candidate proteins is also worthwhile.

When a pathogen infects plants, the avirulence protein is recognized by the R protein, which results in hypersensitive response cell death and prevents the pathogen from infecting the neighboring cells [45]. There are some known genes that have been characterized, such as the HR-like (*HRL*) gene. The *HRL* gene has been reported to be an essential gene in pathogen infection progress, as the mutant plants showed necrotic lesions and embryonic lethal phenotype [46,47,48]. The produce of griffithsin (GRFT) in *Nicotiana benthamiana* leads to necrotic symptoms and HRLI cell death, with high expression of H_2_O_2_ and PR1 [49]. During *Oat dwarf virus* (ODV) infection, the NbFDN1 is an important component induced by H_2_O_2_ expression, resulting in HRLI cell death [50]. The HR involves a complex interaction of host and pathogen factors, so neither the mechanism of HR regulation nor its induction is well understood [51]. Among the 51 interaction proteins, the HRLI protein appeared five times, as suggested its high frequency being pulled out by using SMV P3 as a bait protein, and its important role in SMV infection host plants was chosen for further analysis. The interaction of GmHRLI and P3 was confirmed using BiFC assay, and the interaction fluorescence was detected at the cell periphery as well as at the nucleus. Previous results showed that the SMV P3 promotes GmEF1A nuclear localization, which suggests a parallel mechanism of functions for the HIV-1 Nef protein [35]. Interestingly, the SMV P3 translocated to the nucleus in the presence of GmHRLI in both co-localization assay and BiFC assay. This raises the possibility that GmHRLI might function in the SMV life cycle by becoming involved in the ER stress and having a relationship to GmEF1A.

Expression analysis performed by qRT-PCR showed that the *GmHRLI* gene was induced in both susceptible and resistant plants after SMV infection, but the induction magnitude was higher in susceptible plants. This indicated the involvement of the GmHRLI protein in defense signaling against SMV. While the expression of the *GmHRLI* gene was lower at 12 h than at other time points in the resistance cultivar, the resistance gene might recognize the virus and restrict its replication, and this progress does not require the *GmHRLI* gene. Our experiments also showed that the expression of candidate genes is not consistent with the change trend of viral CP content early in the infection time, which may be because candidate genes, along with multiple other genes, participate in the process of plant disease resistance response.

Here, the *GmHRLI* was shown to be involved in the SMV life cycle, since its higher expression after SMV infection indicated it as an important gene in SMV infection progress by targeting P3. Through identifying potential interactors of P3 in the host plants, our results provide an overall idea of the network of P3 interactors and further clues to study this process, which may provide new insight for breeders to raise resistance plants for the precise function of GmHRLI in the progress of SMV survival in soybean plants. However, in order to assess GmHRLI response to SMV and other potyvirus, further experiments such as virus-induced gene silencing and RNAi silencing are needed.

## 4. Materials and Methods

### 4.1. Plant Growth and Virus Strains

Soybean [*Glycine max* (L.)] cultivar NN1138-2, which is susceptible to SMV, and RN-9, which is resistant to SMV SC15, were grown in an aphid-free greenhouse at 25 °C day and 20 °C night. SMV strain SC15 is one of the most virulent strains in China [8]. When fully expanded, unifoliate leaves come out, and the samples containing the SMV SC15 virus were taken for rub inoculation. The soybean cultivar NN 1138-2 and SMV strain SC15 were provided by Soybean Research Institute of Nanjing Agricultural University. Yeast strain NMY51 (Dualsystems Biotech, Schlieren, Switzerland) is an ideal reporter strain for DUAL membrane screening systems and compatible with most LexA-based yeast two-hybrid systems.

### 4.2. Soybean cDNA Library and Bait Vector Construction

To construct the library, total RNA was isolated from soybean floral after SMV-SC15 strain infection using the RNA TRIzol Reagent (Invitrogen, Carlsbad, CA, USA). mRNA was isolated according to FastTrack® 2.0 Kit’s manufacturer’s procedure. The first-strand cDNA was synthesized from 3 μg of mRNA using attB2-Oligo (dT) primer by SuperScript III reverse transcriptase (Takara Bio, Dalian, China). The complementary strand was synthesized by moloney murine leukemia virus (MMLV) reverse transcriptase (Takara Bio, Dalian, China). cDNA was ligated with three types of attB1 adapters to generate three possible reading frames, which were then introduced to the prey vector pPR3-N-R1R2. The constructs were then electrotransformed into *E.coli* DH10B premium electro cells (Takara Bio, Otsu, Japan) for amplification. The amplified cDNA library plasmid was transformed to yeast NMY51 competent cells, and the transformation efficiency as well as library titer were calculated to make sure the cDNA library was ready for use [52].

For bait vector construction, the P3 insert was cloned from the cDNA using primers with the *Sfi* I adapter listed in Table S2 and purified by agarose gel recovery. Both P3 and bait vector pBT3 were digested by the *Sfi* I restriction endonuclease, which was then ligated together by the T4 DNA ligase. The construct was sequenced to ensure that the insert sequence was correct and confirmed by the *Sfi* I endonuclease, which was digested to check whether it was properly released from PBT3. Then, the sequence-verified bait construct was transformed into NMY51 for control assays.

### 4.3. Yeast Two-Hybrid Assay

The yeast NMY51 competent cells were prepared by the lithium acetate method [53]. The prey vector pPR3-N-R1R2, bait vector pBT3, and recombinant pBT3-P3 were transformed into yeast competent cell NMY51 respectively to check the auto-activation following the yeast transformation protocol, as described by Clontech. Then, they were inoculated on four sorts of nutrition-deficient culture media: the SD^-Trp^ X-α-gal, SD^-Leu^ X-α-gal, SD^-Leu-Trp^ X-α-gal, and SD^-Ade-Trp-Leu-His^ X-α-gal. After air drying, they were cultured at 30 °C for 3–5 days for the observation of their growth.

The cDNA library was transformed into NMY51 competent cells containing plasmid pBT3-P3, and interactors were selected on plates SD^-Leu-Trp-His-Ade^ X-α–gal. Transformed yeast cells (1 mL) were diluted with 0.9% NaCl, and 100 μL of 1:10, 1:100, 1:1000, and 1:10,000 dilutions were spread on 100-mm SD^-Leu-Trp^ plates, respectively, and incubated at 30 °C until clones appeared (4 d). These clones (cfu) were counted to calculate the transformation efficiency and library titer. These steps were performed according to the protocol of yeast mate and plate kit (Clontech USA, Mountain View, USA). Yeast plasmids were isolated from all the positive clones and retransformed into *E. coli* DH5α. Positive clones were amplified by PCR using sequencing primer and tested by electrophoresis on a 1.0% agarose gel. Then, sequences were obtained by Sanger sequencing and identified by a BLAST search. A clone from the bait transformant was mated with a clone from the prey transformant, and grown at 30 °C overnight in 1 mL of yeast extract peptone dextrose broth. The mated clones were selected on SD medium fortified with both tryptophan and leucine to ensure successful mating. Finally, the interacting partners were screened on SD media lacking Trp, Leu, Ade, and His. The pCCW-Alg5 and pAI-Alg5 served as the positive control, and the pCCW-Alg5 and pBT3 served as the negative control.

### 4.4. Bimolecular Fluorescence Complementation and Localization Assays

The BiFC assays were carried out as described before [54]. The interaction proteins were cloned to pSITE-n/cEYFP vectors [55] and transformed into *Agrobacterium tumefaciens* strain LBA4404. Positive agrobacteria that fused with reciprocal halves of EYFP were co-infiltrated into transgenic *N. benthamiana* plants, which expressed the nuclear localized H2B protein tagged with the CFP tag [56]. For localization assay, the P3 protein was cloned to the pEarlyGate 101 vector, and GmHRLI-1 was cloned to pEarlyGate 102. Leaf tissues were sunk in water drop reversal after 2 days and checked by confocal microscopy of PLAPO60XWLSM (NA 1.0) objective. The interaction was confirmed using both combinations of reciprocal nEYFP/cEYFP fusion proteins in two separate experiments (three replicates per experiment). Protein localization assays were done using agro-infiltration of the C-terminal YFP/CFP tagged proteins in transgenic *N. benthamiana* plants expressing RFP-H2B [56].

### 4.5. Sequence Accessions and Phylogenetic Analysis

Database accessions for sequences used here are PvHRLI (AGZ15389), McHRLI (OVA02438), CfHRLI (GAV82386), ToHRLI-1 (PON95086), PaHRLI (PON33343), ToHRLI-2 (PON40966), CcHRLI (OMO54803), McHRLI (OVA02439), CoHRLI-2 (OMO95511), GmHRLI-1 (Glyma19G163200), and GmHRLI-2 (Glyma.03G161600). Sequence alignment and phylogenetic analysis were carried out using the Megalign program in the DNASTAR package [57].

### 4.6. Qutantitative RT-PCR Analysis

Three SMV-infected RN-9 and NN1138-2 leaves were independently collected at 0, 4, 6, 10, 12 h post-inoculation (hpi), as well as the roots, stems, leaves, flowers, and pod from wild-type NN1138-2, and stored at −80 °C. Total RNA was extracted from collected samples using a Simple Total RNA Kit (Tiangen, Beijing, China), and first-strand cDNA was synthesized with oligo (dT) primers using PrimeScript^TM^ И first strand cDNA Synthesis Kit (Takara, Beijing, China), following the manufacturer’s instructions. Based on the GmHRLI gene coding sequence, Primer PREMIER 5.0 software (PREMIER Biosoft International, Palo Alto, California USA) was used to design the gene-specific primers used for quantitative reverse transcriptase PCR (qRT-PCR). Tubulin was used as an internal control to normalize the total amount of cDNA used in each reaction (Primer sequence listed in Table S2). The reactions were carried out with SYBR^®^ Premix Ex Taq^TM^ kit on Roche LC 480II qRT-PCR machine. The mixtures contained 2 μL of sample DNA, 0.4 μL of forward primer, 0.4 μL of reverse primer, 10 μL of 2×SYBR^®^ PremixEx *Taq* and 7.2 μL of ddH_2_O. The PCR program was at 94 °C for 2 min, 35 circles of 95 °C for 5 s, 55 °C for 30 s, 72 °C for 30 s, and 68 °C for 5 min. Expression levels were quantified using the relative quantification 2^−ΔΔCt^ method. Each sample was performed with three biological replicates.

## Supplementary Materials

Supplementary materials can be found at https://www.mdpi.com/1422-0067/20/14/3388/s1.
