# Supplementary material for: Comprehensive Analysis of Soybean Mosaic Virus P3 Protein Interactors and Hypersensitive Response-Like Lesion-Inducing Protein Function
Source: Int J Mol Sci. 2019 Jul 10;20(14):3388. doi: 10.3390/ijms20143388 (PMC6678280; doi:10.3390/ijms20143388)
Supplement: Supplementary file 1 [file ijms-20-03388-s001.pdf]

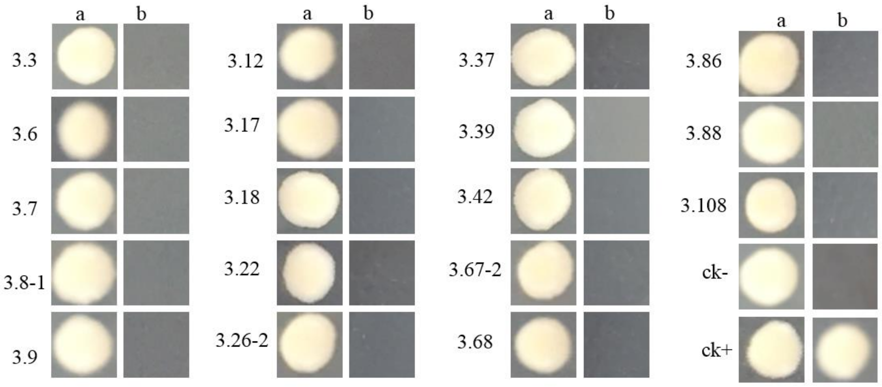

Figure S1. Determination of autonomous activity of candidate proteins. Each candidate protein was co-transformed with pBT3. ck+ is the positive control contained pCCW-Alg5 and pAI-Alg5, ck- is the negative control contained pCCW-Alg5 and pBT3. Yeast cell grown on dropout SD$^{-Leu-Trp}$ medium (a), which corresponded to cells containing the plasmids. Yeast cells grown on dropout SD$^{-Leu-Trp-His}$ medium to determine the autonomous activity (b).

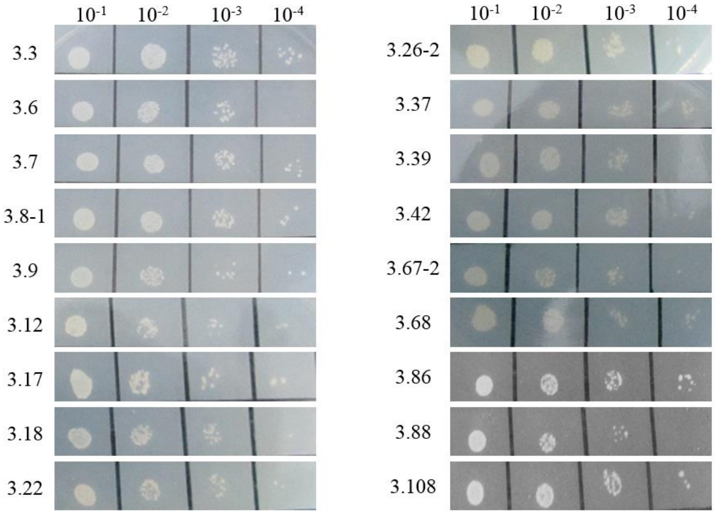

Figure S2. Determination of the interaction strength of each candidate proteins with P3. Transformants containing the bait and prey plasmids were identified based on their growth on dropout the interaction strength was detected on SD$^{-Leu-Trp-His-Ade}$ medium, each drop concentration ranged from 10^{-1}, 10^{-2}, 10^{-3} to 10^{-4}.

Table S1 BLAST analysis of yeast two-hybrid results using P3 as bait to screen the SMV-infected soybean cDNA library

GenBank No.	Protein name	Characteristics	Homology	Clone number	Functional classfication	Cloned sequence/Full length
NM_001362305	Mitochondrial import inner membrane translocase, subunit Tim17/22(Tim)	The pre-protein translocase of the mitochondrial outer membrane (Tom) allows the import of pre-proteins from the cytoplasm.	100%	1	Transport	100%
XP_002523677.1	Zinc transporter (ZIP)	ZRT IRT-like Protein was predicted to be a divalent heavy-metal cations transporter mainly inorganic ion transport and metabolism, alsp the chloroplast precursor.	96%	4	Transport	51.44%
XM_003612815.2	Hypothetical protein aralydraft_914922 (SNARE)	SNARE associated Golgi protein	85%	1	Transport	97%
XP_002865943.1	Hypothetical protein aralydraft_495363 (V-ATPase)	Endoplasmic reticulum-based factor for assembly of V-ATPase	66%	1	Transport	43.98%
XM_003542673.3	Putative plastid triose phophate translocator (PTPT)	arboxylate/Amino Acid AmineTransporter	100%	1	Transport	7.13%
XP_002872953	Hypothetical protein aralydraft_912203 (MIP)	Major intrinsic protein (MIP) superfamily. Members of the MIP superfamily function as membrane channels that selectively transporter,	98%	3	Transport	100%

GenBank No.	Protein name	Characteristics	Homology	Clone number	Functional classfication	Cloned sequence/Full length
		small neutral molecules, and ions out of and between cells.				
NM_001358269.1	Quaporin PIP-type 7a (PIP 7a)	Major intrinsic protein	96%	1	Transport	100%
XM_003555882.3	Sec-independent protein translocase protein (TatC)	Double fertilization forming a zygote and endosperm, intracellular protein transmembrane transport, protein transport by the Tat complex, thylakoid membrane organization.	98%	1	Transport	84%
AAW51884.1	Nitrate and chloride transporter(NCT)	The Major Facilitator Superfamily (MFS) is a large and diverse group of secondary transporters that includes uniporters, symporters, and antiporters. MFS proteins facilitate the transport across cytoplasmic or internal membranes of a variety of substrates including ions, sugar phosphates, drugs, neurotransmitters, nucleosides, amino acids, and peptides.	97%	1	Transport	50.25%

GenBank No.	Protein name	Characteristics	Homology	Clone number	Functional classfication	Cloned sequence/Full length
XM_003519194	Anion transporter 4 (AT4)	The component of a membrane consisting of the gene products and protein complexes having at least some part of their peptide sequence embedded in the hydrophobic region of the membrane.	97%	1	Transport	88%
XM_006593382	SECY/SEC61-α family member	The directed movement of proteins into, out of or within a cell, or between cells, by means of some agent such as a transporter or pore.	96%	1	Transport	75.68%
NM_001254098.2	Plant specific mitochondrial import receptor subunit TOM20	A large complex of the mitochondrial outer membrane that mediates transport of proteins into all mitochondrial compartments.	99%	1	Protein transport	38.07%
XM_003534590.3	Vacuolar-sorting receptor 1 (VSR1)	Protein targeting to vacuole, involved in calcium ion binding.	100%	1	Protein transport	60.75%
XM_006600068.2	Elongation factor 1A (EF1A)	SMV resistance-related protein	98%	5	Defense	100%
NM_001317468.1	Tetraspanin-8-like (TET8)	Defense response to Gram-negative bacterium	99%	1	Defense	100%
XM_003545722.3	PR1a precursor	Contains SCP-like extracellular protein	94%	2	Defense	75.48%

GenBank No.	Protein name	Characteristics	Homology	Clone number	Functional classfication	Cloned sequence/Full length
		domain and belongs to PR-1 like subfamily. The wider family of SCP containing proteins includes plant pathogenesis-related protein 1 (PR-1), which accumulates after infections with pathogens, and may act as an anti.				
XM_006586752.2	Actin-depolymerizing factor 2 (ADF2)	Actin depolymerisation factor/cofilin-like domains; present in a family of essential eukaryotic actin regulatory proteins; these proteins enhance the turnover rate of actin and interact with actin monomers as well as actin filaments.	100%	1	Defense	100%
NM_001248058.1	Protease inhibitor	Predicted to encode a PR (pathogenesis-related) protein. Belongs to the plant defensin (PDF) family. Defense response to fungus,	99%	1	Defense	85.3%
AAC49975.1	Hypersensitive response like (HR-like)	HR-like protein ables to induce HR-like lesions and was associated with the hypersensitive response	68%	4	Resistance related	100%

GenBank No.	Protein name	Characteristics	Homology	Clone number	Functional classfication	Cloned sequence/Full length
		(HR) pathway of defense against plant pathogens.				
Glyma.10G035300	HR-like lesion-inducing (HR-lesion)	HR-like lesion-inducing protein-related	99%	1	Resistance related	100%
AAR26001.1	Endo-1,3-beta-glucanase	Differential Regulation of beta-1,3-Glucanase Messenger RNAs in response to Pathogen Infection	97%	4	Resistance related	100%
NM_001255733.2	Ubiquitin-conjugating enzyme 32 (UBC32)	Involved in plant disease resistance	99%	1	Resistance related	100%
NM_001249173.3	Photosystem II subunit X(PSBX)	A photosystem that contains a pheophytin-quinone reaction center with associated accessory pigments and electron carriers. In cyanobacteria and chloroplasts, in the presence of light, PSII functions as a water-plastoquinone oxidoreductase, transferring electrons from water to plastoquinone, whereas other photosynthetic bacteria carry out an oxygenic photosynthesis and oxidize other compounds to re-	99%	1	Photosynthesis	100%

GenBank No.	Protein name	Characteristics	Homology	Clone number	Functional classfication	Cloned sequence/Full length
		reduce the photoreaction center.				
XP_002521096.1	Cytochrome B5 isoform 1(Cyt B5)	Cytochrome b5-like Heme/Steroid binding domain.	99%	2	Photosynthesis	100%
ABC46708.1	Photosystem II subunit R (PSBR)	Encodes for the 10 kdaPsbR subunit of photosystem II (PSII). This subunit appears to be involved in the stable assembly of PSII, particularly that of the oxygen-evolving complex subunit PsbP. Mutants defective in this gene have reduced amounts of subunits PsbP and PsbQ in PSII. In turn, assembly of PsbR is dependent on the presence of PsbJ.	90%	6	Photosynthesis	0%
XM_006592304.2	Tetratricopeptide repeat (TPR)-like	Encodes one of the 36 carboxylate clamp (CC)-tetratricopeptide repeat (TPR) proteins (Prasad 2010, Pubmed ID: 20856808). Functions as a chaperone receptor at the chloroplast outer envelope, mediating Hsp70-dependent protein	99%	2	Photosynthesis	19.33%

GenBank No.	Protein name	Characteristics	Homology	Clone number	Functional classfication	Cloned sequence/Full length
		targeting to chloroplasts. It has been localized to the ER membrane, interacts with the Sec translocon, and has a potential function in post-translational protein transport into the ER. The mRNA is cell-to-cell mobile.				
XP_002523677.1	Copper response defect1 (CRD1)	Encodes a putative ZIP protein with varying mRNA accumulation in leaves, stems and roots. Has a consensus carboxylate-bridged di-iron binding site. The mRNA is cell-to-cell mobile.	88%	1	DNA binding	57.25%
XM_006605908.2	Stress enhanced protein 1(SEP1)	Encodes a stress enhanced protein that localizes to the thylakoid membrane and whose mRNA is upregulated in response to high light intensity. It may be involved in chlorophyll binding.	94%	1	Chlorophyll binding	80.89%
NM_001252782	Reticulon family protein	VIRB2-interacting protein 2	94%	1	Protein binding	79.22%
XM_003543121.3	60S ribosomal protein L18a	Integral component of membrane	99%	1	Protein binding	100%

GenBank No.	Protein name	Characteristics	Homology	Clone number	Functional classfication	Cloned sequence/Full length
NM_001248577.1	Metallothionein 2B (MT2B)	Interacting selectively and non-covalently with any metal ion. Cysteine-rich protein with copper-binding activity.	100%	1	Biological Process	100%
XM_006596365.2	Selenoprotein-related (SELT)	Selenoprotein. Rdx type; Functions in: selenium binding; Involved in: cell redox homeostasis; Located in: endoplasmic reticulum, plasma membrane.	98%	2	Cell redox homeostasis	66.63%
NM_001255733.2	Ubiquitin-conjugating E2 (UBC2)	The protein ubiquitination, has ATP binding, ubiquitin protein ligase activity, ubiquitin protein ligase binding and ubiquitin-protein transferase activity.	100%	4	Protein degradation	22.01%
XM_014773853.1	Ankyrin like protein(ALP)	Encodes a member of the ankyrin repeat protein. Localized in the endoplasmic reticulum.	68%	1	Protein interaction	47.45%
XM_003522001	Sec61 translocon complex (Sec61TC)	A translocon complex that contains a core heterotrimer of conserved alpha, beta and gamma subunits, and may contain additional proteins (translocon-associated proteins or traps); in	99%	1	Protein synthesis	100%

GenBank No.	Protein name	Characteristics	Homology	Clone number	Functional classfication	Cloned sequence/Full length
		budding yeast the core proteins are Sec61p, Sbh1p, and Sss1p. The Sec61 translocon complex functions in cotranslational and posttranslational translocation events.				
AK244083	40S ribosomal protein S19	The cellular metabolic process in which a protein is formed, using the sequence of a mature mRNA molecule to specify the sequence of amino acids in a polypeptide chain. Translation is mediated by the ribosome, and begins with the formation of a ternary complex between aminoacylated initiator methionine tRNA, GTP, and initiation factor 2, which subsequently associates with the small subunit of the ribosome and an mRNA. Translation ends with the release of a polypeptide chain from the ribosome.	98%	1	Basal	51.85%

GenBank No.	Protein name	Characteristics	Homology	Clone number	Functional classfication	Cloned sequence/Full length
XM_006574623.2	Chaperone-like protein of POR1	Unknown	99%	1		77.78%
XM_014776821.1	Trab family protein	Unknown	98%	1		93%
KM407510	Unknown	Unknown	96%	1		100%
BT091280.1	Unknown	Unknown	99%	1		100%
AK245224	Unknown	Unknown	99%	1		0.01%
ACU20654.1	Unknown	Unknown	97%	1		100%
BT096280	Unknown	Unknown	99%	1		100%
AK287352	Unknown	Unknown	99%	1		100%
EF210198	Unknown	Unknown	88%	1		16.89%
AK245441.1	Unknown	Unknown	100%	1		55.80%
KF170378	Unknown	Unknown	90%	1		75.36%
ACU23929	Unknown	Unknown	90%	1		89.47%
KU904418	Unknown	Unknown	94%	1		0%
XM_003553097	Unknown	Unknown	100%	1		100%

Table S2. Primer sequence

Gene name	Primer sequence （5′→3′）
GmHRLI F	TTTGTACAAGAAAGCTGGGTCATAGGTTTTTGTTTTGGGAACCTTC
GmHRLI R	AGTTTGTACAAAAAAGCAGGCTTCATGGCGTTCTCTTCTTTTCTCG
qHRLI F	TCTAAAGCTGGCTTTCA
qHRLI R	GGAGTAGCAATCATTTGA
SMV-P3 F	ATTAACAAGGCCATTACGGCCGGGGAAGTGCAACAAAGGATGA

SMV-P3 R	AACTGATTGGCCGAGGCGGCCCCCTGTGCGGAGACATCTTCTGA
Tubulin F	GGAGTTCACAGAGGCA GAG
Tubulin R	CACTTACGCATCACATAGCA
pPR3-N F	GTCGAAAATTCAAGACAAGG
pPR3-N R	AAGCGTGACATAACTAATTAC
